# Development of Cd (II) Ion Probe Based on Novel Polyaniline-Multiwalled Carbon Nanotube-3-aminopropyltriethoxylsilane Composite

**DOI:** 10.3390/membranes11110853

**Published:** 2021-11-04

**Authors:** Raja S. Alruwais, Waheed A. Adeosun, Amjad E. Alsafrani, Hadi M. Marwani, Abdullah M. Asiri, Imran Khan, Mohammad Jawaid, Anish Khan

**Affiliations:** 1 Chemistry Department, Faculty of Science, King Abdulaziz University, Jeddah 21589, Saudi Arabia; rajaotb@gmail.com (R.S.A.); dsnwaheed1@gmail.com (W.A.A.); Amjad.chem@hotmail.com (A.E.A.); aasiri2@kau.edu.sa (A.M.A.); 2 Department of Chemistry, Faculty of Science and Humanities, Shaqra University, Dawadmi 11911, Saudi Arabia; 3 Center of Excellence for Advanced Materials Research, King Abdulaziz University, Jeddah 21589, Saudi Arabia; 4 Department of Chemistry, College of Science, University of Jeddah, Jeddah 21589, Saudi Arabia; 5 Applied Sciences and Humanities Section, University Polytechnic, Faculty of Engineering and Technology, Aligarh Muslim University, Aligarh 202002, India; imrannano@gmail.com; 6 Laboratory of Biocomposite Technology, Institute of Tropical Forestry and Forest Products (INTROP), University Putra Malaysia, UPM, Serdang 43400, Selangor, Malaysia

**Keywords:** linear sweep voltammetry, heavy metal detection, environmental pollution, cadmium ion detection, electrochemical sensors, potential toxic metals

## Abstract

Cadmium belongs to the group of potentially toxic metals that have high health and environmental significance. Due to its adverse effects on the environment, this study develops an effective electrochemical sensor for detecting a polyaniline-multiwalled carbon nanotube-3-aminopropyltriethoxysilane (PANI-MWCNT-APTES) substrate cast on the GCE. The as-prepared PANI-MWCNT-APTES was prepared by a wet chemical method, and its formation was investigated using several techniques. As a result, the prepared material exhibited a limit of detection of 0.015 µM for cadmium ions (Cd^2+^) in the linear dynamic range of 0.05 µM to 50 µM. Furthermore, the PANI-MWCNT-APTES-modified GCE current response was stable, repeatable, reproducible, and short. In addition, PANI-MWCNT-APTES/GCE was harnessed for the first time for cadmium detection in real water samples, and the result was satisfactory. Therefore, the recorded results suggest that the newly designed PANI-MWCNT-APTES is a promising material for detecting Cd in the near future for human health and environmental protection.

## 1. Introduction

The world at large has consistently witnessed ever-increasing environmental pollution over the years, especially in the aquatic environment (water bodies), due to growing industrial establishment and operations. The effluents arising from industrial operations contain many toxic chemicals and potentially toxic metals (PTMs), which include metal ions such as cadmium. Cd (II) is highly generated from industrial processes such as textile operation, tanning, aluminum extrusion, agricultural processes, etc. [1,2,3]. The resultant effluents from the aforementioned industrial processes enter oceans and rivers and even percolate into underground aquifers [4]. Through this, Cd^2+^ interferes with aquatic lives and even affects humans through the oral route [5,6]. The toxicity of Cd^2+^ lies in its ability to bioaccumulate on living organisms, from trace concentrations to much higher concentrations [5]. At a certain concentration, Cd^2+^ becomes highly toxic to humans and another living organism in the biota. The permissible limit of Cd^2+^ in potable water and surface water indicated by the World Health Organization are 3 ng/mL and 5ng/mL, respectively [7,8]. A higher concentration of Cd^2+^ has been implicated in several health problems including kidney dysfunction, cancer, a blood disorder, and bone pain [9,10,11]. Due to its health significance and threat, there is a need for research on developing a sensitive and effective method of Cd^2+^ detection and monitoring in the environment, especially in drinking water and oceans. In the past and even till now, several efforts have been expended on developing methods of Cd^2+^ detection. These methods include atomic absorption spectrophotometry [12], inductively coupled plasma spectrophotometry [13], UV spectrophotometry [14], etc. These methods are sensitive and suitable for Cd^2+^ detection; however, they suffer setbacks in their high cost of analysis, cumbersomeness in sample preparation and equipment operation, high time consumption, unsuitability for in situ analysis, etc. Therefore, to address this problem, a fast, reliable, and sensitive method is required. An electrochemical method fits this, as it is speedy, reliable, suitable for in situ analysis, and cheap [15,16]. Therefore, the current study employs the electrochemical method for Cd^2+^ detection in water. 

Previously, researchers have explored the detection of Cd^2+^ using the electrochemical method, but the methods suffer some setbacks such as result instability and high limits of detection. For instance, Hassanpoor et al., in their study, employed MnO2-reduced graphene oxide (rGO) nanocomposite Cd^2+^ sensing in an aqueous medium. The developed sensor displayed a high sensitivity with a relatively low limit of detection (1.12 µg/L). However, the current response of the developed sensor suffers instability in the presence of likely interferents, especially at high concentrations [17].

Wang et al. reported the detection of cadmium ion in wastewater using a thiacalix [4] arene (TC4A)-modified electrode. The electrochemical detection of Cd^2+^ was carried out using differential pulse anodic stripping voltammetry (DPASV). They reported a reduction in current responses in the linear dynamic range of 0.1–1 mg/L with a limit of detection of 4µg/L. The developed Cd ion sensor was reported to give good sensitivity and stability in water. However, the linear response of the sensor was deficient [18].

A lot of study is conducting on composite meterials for different applications because of high increase properties after making composite by involving individual initiators quality too [18,19,20,21,22,23,24,25,26,27,28]. Therefore, to improve the current response towards Cd^2+^ concentration, especially in terms of response stability, we explored a conducting polymer-based composite-coated electrode material. Specifically, the sensing material based on polyaniline-multiwalled carbon nanotube-3-aminopropyltriethoxysilane (PANI-MWCNT-APTES) composite was selected. The choice of this material was based on the excellent properties of the constituent of the composite. For instance, polyaniline is a conducting polymer with excellent properties such as high conductivity due to its delocalized pi-electron, high catalytic property, and high chemical stability [29,30]. In addition, the multiwalled carbon nanotube is a highly conductive and catalytic material, and it has equally good mechanical and chemical stability [31,32]. In addition, compositing MWCNT to PANI provides more active nucleation sites for PANI, excellent electron transfer, and good mechanical stability.

Therefore, compositing these materials is expected to result in a highly conductive, catalytic, and chemically stable material, which would have strong potential to act as electrocatalysts for electro-oxidation or for the reduction of heavy metal ions in an aqueous medium. 

As far as we know, this study reports the synthesis of PANI-MWCNT-APTES composites for application as a Cd^2+^ electrochemical probe for the first-time. 

## 2. Experimentation

### 2.1. Reagents Used

The reagents utilized for this research includes aniline monomers, multiwalled carbon nanotubes, potassium persulfates, hydrochloric acid, 3-aminopropyltriethoxysilane-98%, an acetate buffer, potassium ferricyanide, and deionized water obtained from Milli-Q plus system. All of the reagents were procured from Sigma Aldrich (St. Louis, MO, USA) USA and were used without any further purification. 

### 2.2. Instrumentation

In this study, several techniques were used for material synthesis, characterization, and application. Among the instruments used are a Fourier Transform infrared spectrometer, FTIR (Perkin Elmer 2000, Waltham, MA, USA), a field emission scanning electron microscope, FESEM (JEOL JSAM 6300, Jeol, Tokyo, Japan), an X-ray diffraction spectrometer, XRD (X-Max Oxford Instruments, Abingdon, UK), an X-ray photoelectron spectrometer, XPS (Thermo Scientific Waltham, MA, USA), a thermogravimetric analyzer, TGA (Mettler Toledo, Greifensee, Switzerland), and an electrochemical work station (Autolab Potentiostat—PGSTAT302N-AUT85887) controlled by Nova 2.0 software. The electrochemical workstation comprises a working electrode (glassy carbon electrode—modified and bare), a reference electrode (Ag/AgCl—in 3M KCl), and a 0.5 mm wide platinum counter electrode. The electrical conductivity measurement was carried out using a four-probe method on press pallets of the PANI@MWCNT-APTESP using the DMV-001 digital multimeter (SES instrumentation, PVT. LTD., Roorkee, India).

### 2.3. Synthesis of PANI-MWCNT-APTES Composite

Polyaniline was prepared by the conventional chemical oxidation method, where the oxidation of aniline monomer was achieved by potassium persulfate (K_2_S_2_O_8_). At first, a varying concentration of 0.1 M aniline monomer was prepared in 1 M hydrochloric acid to form aniline hydrochloride. Then, the oxidizing agent, 0.1 M K_2_S_2_O_3_, was added gently to a varying volume of 0.1 M aniline (3, 5, and 7 mL dissolved in 100 mL 0.1 M K_2_S_2_O_8_ under vigorous stirring for 2 h at 0–4 °C. The incorporation of MWCNTs into PANI was performed using a mechanical method. Initially, before crosslinking to PANI, MWCNT-APTES was synthesized. Specifically, 200 mg of MWCNTs was dissolved in 50 mL ethanolic solution (65% v/v) and was ultrasonicated for 1 h. Subsequently, 2 mL of the APTES solution was added to the mixture. Finally, the mixture (APTES-MWCNTs) was added to the PANI matrix under vigorous stirring for 1 h to crosslink with PANI. The obtained PANI-MWCNT-APTES was then annealed at 75 °C overnight. The as-prepared PANI-MWCNT-APTES composite was then obtained and used for the remaining studies.

### 2.4. Fabrication of Electrodes for Sensing Application

The synthesized PANI-MWCNT-APTES composite was applied as the substrate on the surface of GCE for sensing application. Before the sensing application, the unmodified glassy carbon electrode (b-GCE) was thoroughly cleaned under sonication. To remove the materials adhered onto the b-GCE surface, it was electrochemically cleaned by a cyclic voltammetric sweep in 0.25 M H_2_SO_4_. The clean b-GCE was then collected, and 1 mg of the composite (PANI-MWCNT-APTES) was cast on it with the aid of 0.1 µL of nafion. The coated PANI-MWCNT-APTES-modified GCE was then put in the oven to dry at 50 °C for 30 min. The as-prepared PANI-MWCNT-APTES-GCE was then kept and used for further experiment.

### 2.5. Electrochemical Studies

The electrochemical studies of interest to this work include impedance spectroscopy, cyclic voltammetry, and linear sweep voltammetry. The frequency was set from 0.1 Hz to 100 kHz at 0.005 V amplitude and +0.2 V DC potential for the impedance spectroscopy. Cyclic voltammetry was used for electrochemical characterization using a potential window of −1 V to 1.2 V, a scan rate of 50 mV/s, and a step potential of 0.008 V. The LSV was performed using a potential window of −1 V to 1 V, a scan rate of 75 mV/s, and a modulation time of 0.024 s.

For the electrical conductivity measurement, 200 mg of the as-prepared PANI-MWCNT-APTES was finely ground to make pellets made with the aid of a hydraulic pressure machine operated at a pressure of 5 KN imposed for 20 min. A screw gauge determined the thickness of the pellets. The pellets made were subsequently used for electrical conductivity measurement at room temperature using the four-probe method.

### 2.6. Real Sample Analysis

In order to evaluate the efficacy of the developed probe for Cd^2+^ detection, real samples are collected and used for real-life Cd^2+^ determination. Seawater (obtained from Red Sea Jeddah, KSA) and underground water were used as real environmental samples. All of the samples were collected according to standard procedures and stored at 5 °C. In order to remove the matrix effect, a 50-fold dilution with acetate buffer was performed on the water samples. After that, a prepared standard solution of Cd^2+^ was spiked into the mixture, and the spiked concentration percentage recovery was determined.

## 3. Results and Discussions

### 3.1. PANI-MWCNT-APTES Composite’s Morphological Investigation

**Figure 1:**
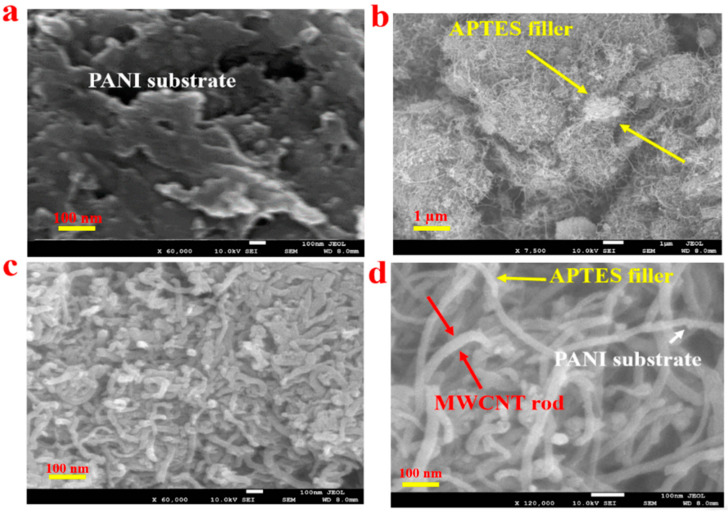
Scanning electron microscope images of (**a**) the PANI matrix, (**b**) the PANI-APTES composite, and (**c**) PANI-APTES-MWCNTs and (**d**) a magnified image of PANI-APTES-MWCNTs.

The morphological property of the as-prepared PANI-MWCNT-APTES composite was studied through scanning electron microscopy (SEM). The obtained SEM images are given in Figure 1. 

From the FESEM image observation, the PANI synthesized is a semi-amorphous porous layer. The incorporation of APTES into the PANI porous layers could be observed in Figure 1b. It could be suggested that APTES, in this case, acts as a filler to the PANI matrix. Further doping with a multiwalled carbon nanotube is evident in Figure 1c,d, where the nanorod of multiwalled carbon nanotube reinforced the PANI-APTES composite. Thus, the obtained images from the FESEM analysis are suggestive of a composite material.

### 3.2. Elemental Study and Structural Investigation of PANI-MWCNT-APTES

**Figure 2:**
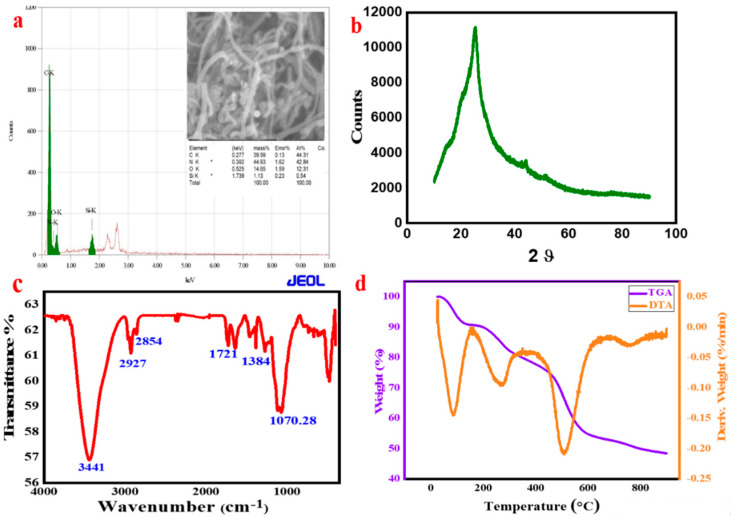
(**a**) XEDS spectrum of PANI-MWCNT-APTES, (**b**) XRD spectrum, (**c**) FTIR spectrum, and (**d**) TGA and DTG recorded for PANI-MWCNT-APTES.

The elemental composition of the synthesized composite was investigated using energy-dispersive X-ray spectroscopy (XEDS). The obtained result is displayed in Figure 2a. The result indicates that the elements predominately present in the as-prepared samples were carbon, nitrogen, oxygen, and silicon, which is consistent with the actual constituent of PANI-MWCNT-APTES.

The structural property of the synthesized *PANI-MWCNT-APTES* was assessed by X-ray diffraction spectroscopy. The obtained XRD spectrum is given in Figure 2b. The diffraction peaks were recorded in the range of 10° to 80°. The obtained prominent peaks at 14.5°, 20.2°, and 25° are peculiar to PANI, and the peaks are inconsistency with the (011), (020), and (200) planes [33,34]. The incorporation of MWCNT in the PANI matrix can be seen at a diffraction angle of 25.2°, inconsistent with the (002) phase [35]. The peak at 20° is peculiar to PANI, and the angle diffraction peak at 24° could be related to the silica group attached to the composite [36]. The obtained XRD spectrum revealed the non-crystallinity of the synthesized PANI-MWCNT-APTES.

### 3.3. Functionalities Investigation Using Infrared Spectroscopy (FTIR)

FTIR was conducted for the identification of the functionality in the as-prepared composite. The FTIR spectrum obtained was in the wavenumber range of 500 cm^−1^ to 4000 cm^−^^1^, as given in Figure 2c. The observed peaks at 2927 cm^−1^ and 2854 cm^−1^ are attributable to the methyl–CH_3_ and –CH_2_ groups associated with PANI and MWCNT [37]. The peak at 1721 cm^−1^ is linked to the C–N stretch in PANI. The N–H stretch in the PANI ring and APTES gives rise to the strong absorption peak observed at 3441 cm^−1^ [38,39]. The C–N vibration that could arise due to crosslinking between APTES/PANI and MWCNT might not be unconnected to the peak observed at 1650 cm^−1^. In addition, the C=C in the conjugated PANI ring (benzenoid) could be linked to the peak at 1384 cm^−1^. The incorporation of APTES into PANI-MWCNT is confirmed by the peaks observed at 1100 cm^−1^ to 1070 cm^−1^, which is peculiar to the Si–O–Si and Si–O–C stretching vibration, respectively [40]. Therefore, the obtained FTIR results are suggestive of successful synthesis of PANI-MWCNT-APTES.

### 3.4. Thermogravimetry Analysis (TGA)

TGA is an important technique used to establish the stability of materials. The result of the TGA analysis is presented in Figure 2d. It could be observed that a weight loss of about 60% is recorded after 800 seconds. The material displayed good stability at an even higher temperature, which might be traced to the effect of MWCNT crosslinking the polymeric composite [41,42]. The differential thermal analysis (DTA) curve revealed three major endothermic reaction stages, namely at 103°, 280°, and 520 °C. The obtained result suggests an endothermic reaction.

### 3.5. Binding Energy

**Figure 3:**
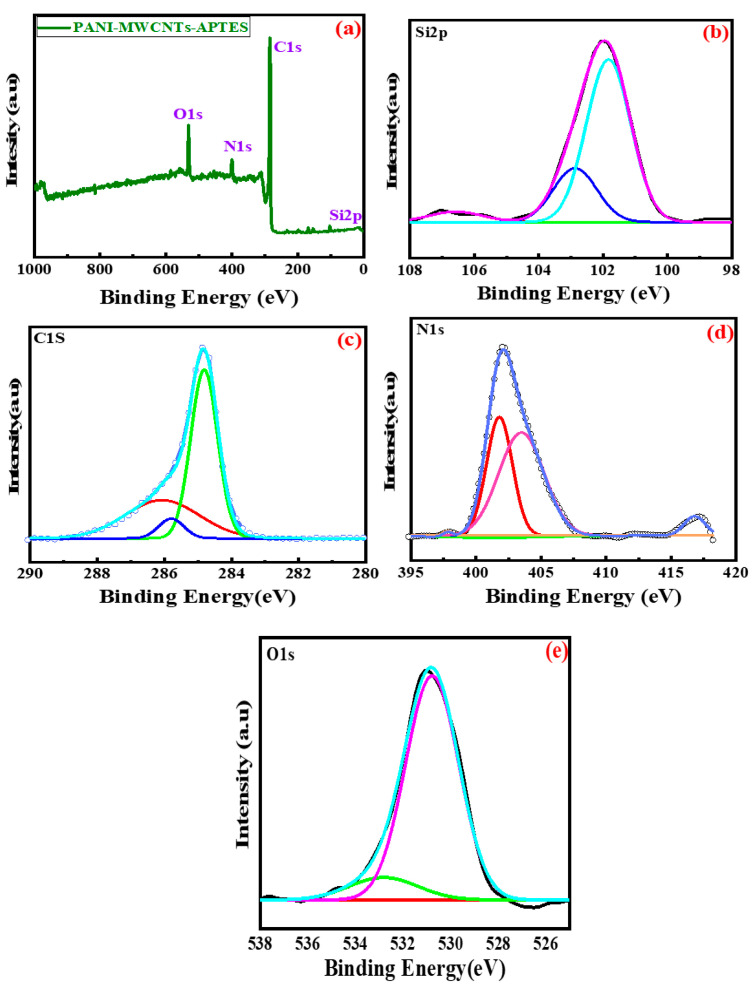
XPS spectrum of PANI-MWCNT-APTES: (**a**) Full spectrum. (**b**) Deconvoluted peak for Si. (**c**) Deconvoluted peak for C. (**d**) Deconvoluted peak for N. (**e**) Deconvoluted peak for O.

The binding energy of the constituent elements in the as-prepared polymeric composite is presented in Figure 3. The binding energy is obtained through XPS. Similar to XEDS, the obtained results confirm the presence of carbon, nitrogen, oxygen, and silica. The absence of other peaks indicates the successful completion of the chapter. The full XPS spectrum is illustrated in Figure 3a. The deconvolution of peaks was carried out to reveal a different chemical state of the individual constituent’s element present in the sample. The C 1s peak is deconvoluted into two peaks, namely at 284.7 eV and 285.5 eV (Figure 3c). The binding energy of C 1s gives a peak at PANI. The other peaks at 284.5, 285.8, 288.78, and 290.58 eV could be linked to C–C, C–O, C–H and –COOH, and π–π* [43].

The peak at around 530 eV is attributable to the oxygen peak. It was deconvoluted into two peaks, 531 eV and 533.2 eV belonging to C-O bond of carboxylic acid in MWCNT and Si-O of triethoxylsilane constituent (Figure 3e). Finally, the peak observed at 100 eV revealed peaks at 102 and 103 eV attributable to Silica. (Si-O) of the triethoxylsilane (Figure 3b) [44]. Nitrogen also exhibits two peaks when deconvoluted, giving rise at 402 eV and 416 eV (Figure 3d). This peak, as indicated earlier, could be linked to N-H strong stretch of PANI and C-N of PANI/MWCNT [26].

### 3.6. Electrical Conductivity of The PANI-MWCNT-APTES

The PANI-MWCNT/APTES nanocomposite pellet’s electrical resistivity was calculated by a sample conductivity test using the four-probe process. The electrical conductivity was determined using the following equations described in Supplementary S1 [45].

**Figure 4:**
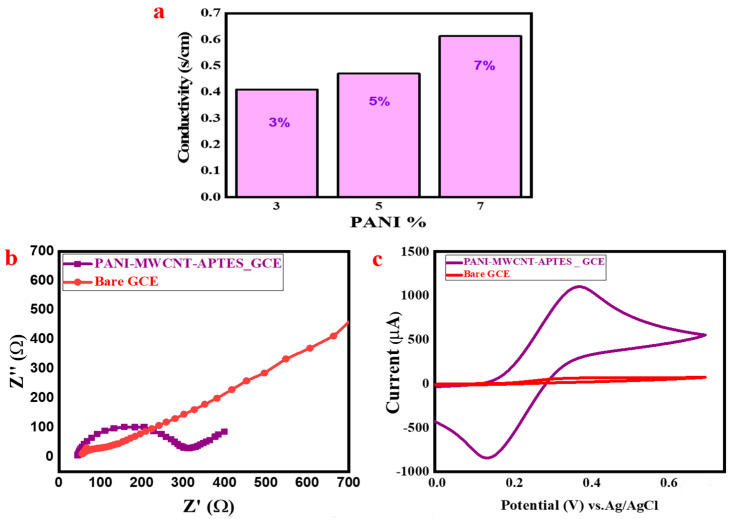
(**a**) A chart showing the electrical conductivity of the as-prepared PANI-MWCNT-APTES. (**b**) The obtained Nyquist plot for PANI-MWCNT-APTES. (**c**) The obtained CV in a ferricyanide couple (1 mM Fe (CN)_6_^3-/4-^ prepared in 0.1 M KCl).

At room temperatures, the electrical conductivity variation (σ) of the as-prepared PANI-MWCNT/APTES nanocomposite (3%, 5%, and 7% PANI concentration constituents) is presented in Figure 4a and Supplementary Table S1. The critical concentration of PANI in PANI@MWCNT/APTES for the highest conductivity was found (7%), as shown in (Figure 4a). Therefore, the composite using higher PANI contents has lower electrical resistivity at 1.63 Ω cm and higher conductivity, calculated by the formula described in Supplementary S1.

### 3.7. Electrochemical Behavior Investigation

The EIS and CV behavior in potassium ferricyanide couples was studied, as was the electrochemical behavior of the as-prepared material (PANI-MWCNT-APTES). The result of the EIS study is given in Figure 4b. The EIS was obtained from the plot of imaginary impedance and against the real impedance. The plot of these two is expressed as the Nyquist plot. The semi-circle of the Nyquist plot refers to the charge transfer resistance (Rct), a function of the conductivity/resistivity. The smaller the Rct, the better the mobility of the charge/ion in the electrolyte [44,46]. For this study, the obtained Rct for PANI-MWCNT-APTES is 131 Ω, while that of the bare electrode was 271 Ω (Supplementary Figures S1 and S2). Therefore, the value obtained for PANI-MWCNT-APTES-modified GCE is smaller than that for the bare one and therefore has less resistance.

In addition, the cyclic voltametric response in the 1 mM Fe (CN)_6_^3−/4−^ solution is given in Figure 4c. The PANI-MWCNT-APTES-GCE displayed a higher oxidation (1 mA) with a smaller peak-to-peak potential (395 mV). These phenomena indicate a faster electrochemical reaction on the PANI-MWCNT-APTES surface compared with bare GCE.

### 3.8. Electrochemical Sensing of Cd^2+^

The response of the fabricated material (PANI-MWCNT-APTES) coated on glassy carbon electrode towards a Cd^2+^ ion was examined using electrochemical techniques: cyclic voltammetry and linear sweep voltammetry. 

#### 3.8.1. Control Study

**Figure 5:**
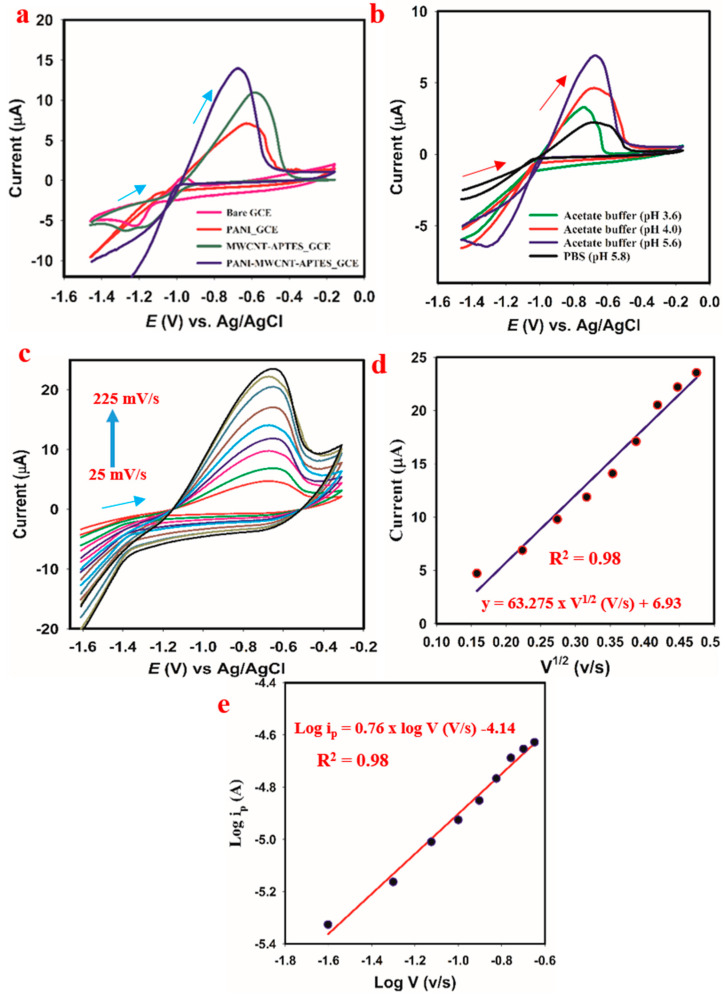
Electrochemical sensing responses: (**a**) effect of pH medium on the reduction in the current response exhibited by PANI-MWCNT-APTES in 15 µM CdSO_4_. (**b**) Control study in 50 µM CdSO_4_. (**c**) The obtained CV upon variation of the scan rate in 30 µM CdSO_4_ (**d**) Linear plot of the scan rate variation against current. (**e**) Linear plot of the logarithm for a reduction in current versus the logarithm of the scan rate.

To start with, a controlled study was conducted to confirm that the recorded current response was due to the effect of PANI-MWCNT-APTES. The obtained result for the control experiment is presented in Figure 5a. It could be observed that, in the absence of PANI-MWCNT-APTES on the GCE, this was a tiny reduction in the current at a higher voltage, at −0.64 V. However, PANI-MWCNT-APTES-modified GCE displayed a sharp higher reduction peak at the reduced potential (−0.7 V). Moreover, the individual effect of the constituent PANI-MWCNT-APTES composite was investigated. As a result, PANI-only-modified GCE gave less reduction in the current at -0.7 V than PANI-MWNT-APTES. This indicates that the composition of PANI with MWCNT-APTES drastically improved the catalytic performance of PANI, thereby leading to its effectiveness in catalyzing the reduction of Cd^2+^ to zerovalent Cd.

#### 3.8.2. PH Optimization

To start with, the effect of pH on the reduction in the current response exhibited by PANI-MWCNT-APTES towards Cd^2+^ ions was studied. The obtained result is presented in Figure 5b. The optimal result (highest reduction in the current) was obtained at pH 5.6 (HAc–NaoAc buffer), while the smallest current response was observed in the acidic phosphate buffer (pH 5.8). This study was conducted in an acidic medium because, in the alkaline medium, cadmium was precipitated with a clear visible white precipitate. This resulted in low or no reduction in the current (Figure not shown). The reason for the low reduction in the current could be associated with possible electrode surface fouling. Moreover, the solubility of metal ions decreases with an increase in pH medium, which could be why, at the essential medium, the concentration of Cd^2+^ reduced [47]. Therefore, the optimal pH was 5.6, and this was maintained for the remaining experiment.

In addition, the scan rate effect on the reduction in current response of PANI-MWCNT-APTES was also studied. The purpose of the scan rate study was to determine whether the electrochemical reduction of Cd^2+^ ion on the GCE substrate is a diffusion control or adsorption-controlled. Usually, a linear dependence of the current on the scan rate plus having the slope of the current against the scan rate in the range of 0–0.5 are indications of diffusion-controlled reactions. For this study, the current response increased with an increase in the scan rate with a correlation of 0.98 Equation (1). The correlation value (0.98) might suggest a non-linear relationship, and this would result in an adsorption-controlled reaction.

ip (µA) = 63.275 × ʋ^1/2^ + 6.9337 (R^2^ = 0.98) (1)

As given in [48,49], the slope of the logarithm of reduction in the current plot against the logarithm of the scan rate that fell below 0.5 indicates a diffusion-controlled reaction. In contrast, the one close to 1 indicates an adsorption-controlled process. Thus, for this study, the slope obtained for the plot of the current reduction logarithm against the scan rate logarithm is 0.765 (Equation (2)).

Log ipc (A) = 0.765 × log v (V/s) − 4.13.(2)

This indicates that the reduction process of Cd^2+^ ion on the PANI-MWCNT-APTES-modified GCE was proceeded by the adsorption-controlled process. 

#### 3.8.3. Effect of Increase in Cd^2+^ Concentration on the Current Response

The effect of varying concentrations of Cd^2+^ ion was investigated using linear sweep voltammetry. Linear sweep voltammetry was employed for this study because it is very accurate and is best suited for irreversible reactions [50,51]. This technique is based on the mechanism of measurement of the current given when the potential between the working electrode and the reference electrode varied linearly with time. The mechanism of Cd^2+^ detection is through the reduction and deposition of zerovalent Cd on the surface PANI-MWCNT-APTES-modified GCE. This type of reaction is irreversible, as presented in Equation (3). 

**Figure 6:**
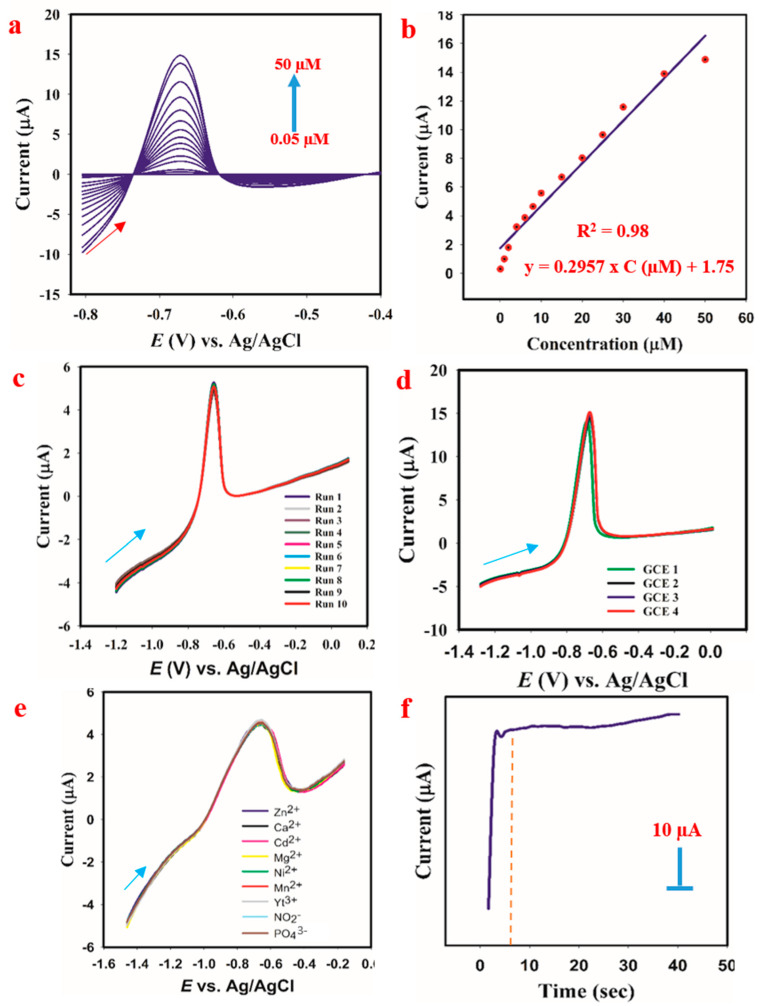
(**a**) Effect of varying the Cd^2+^ ion concentration. (**b**) Linear plot of current response against Cd^2+^ concentration. (**c**) Repeatability test. (**d**) Reproducibility test. (**e**) The effect of interferents on current response. (**f**) The recorded response time.

The obtained linear sweep voltammogram is given in Figure 6a. The result indicates a linear increase in the current reduction response with an increase in the Cd^2+^ ion concentration. This reduction in current indicates the reduction of Cd^2+^ to zerovalent Cd, as suggested in Equation (3).

(3) $$Cd^{2+}+2e^{-}\;\to Cd$$

The slope of the calibration showed a linear relationship between the two parameters (concentration and current) with a correlation of 0.98%. 

#### 3.8.4. Evaluation of Sensor’s Performance

The developed method of Cd^2+^ ion detection based on linear sweep voltammetry was assessed on some analytical performance parameters such as detection limit (LOD), the limit of quantification (LOQ), sensor’s sensitivity, linear dynamic range (LDR), full dynamic range (FDR), and response time.

The LOD was calculated using the following formula (Supplementary S2):

*LOD* = [3 *× Sd of the blank*]/[*slope of the calibration plot*]

The obtained value was 0.0155 µM, as shown in Supplementary S2.

The LOQ was calculated using the following formula:

*LOQ* = 10 × [*standard deviation of the blank*]/[*slope of the calibration plot*]

The obtained value was 0.0517 µM, as shown in Supplementary S2.

The LDR indicates the range at which there is linear dependence of the current on the concentration. This ranged from 0.05 µM to 50 µM. However, the FDR indicates the full concentration range at which current response to the change in concentration was noticed. For this study, this ranged from 0 to 50 µM.

The sensitivity of PANI-MWNT-APTES-modified GCE to Cd^2+^ ions was obtained using the following formula:

*Sensitivity = slope of the calibration/surface area of the GCE*

The obtained value was 4.237 µA µM^−1^ cm^−2^, as shown by Supplementary S2.

Response time is a significant parameter in sensing applications because it indicates the time frame for the electrochemical process. Usually, a sensor with a fast response time is desirable for in situ analysis [52]. For this study, the response time recorded was 9 s (Figure 6f).

The stability of the current response of PANI-MWCNT-APTES was investigated by repetitive measurement techniques (repeatability and reproducibility). While repeatability indicates the accuracy of a method, reproducibility shows the precision of the sensor’s performance. For this study, ten consecutive measurements at the same experimental condition were conducted, and the obtained result is given in Figure 6c. The RSD of the mean value was 0.7%. For reproducibility, four different electrodes were modified with PANI-MWCNT-APTES, and their current response taken at different times was taken (Figure 6d). The RSD of the current responses of the four electrodes was 2.8%.

#### 3.8.5. Interferents’ Effect on the Current Response

Usually, metal ions with similar charges tend to interfere with the redox reaction of each other. For instance, Cd^2+^ has two positive charges (2+) and is prone to interferents from metal ions with similar charges. Therefore, likely interfering ions mostly considered for this study are Mg^2+^, Mn^2+^, Ni^2+^, Ca^2+^, Zn^2+^, Yt^3+^, NO_2_^−^, and PO_4_^3−^. The obtained result is given in Figure 6e. It could be observed that, upon spiking of these likely interferents, the reduction current of Cd^2+^ ions was reduced slightly. However, the relative standard deviation (RSD) of the mean value of Cd^2+^ ions is less than 5%. Therefore, the response of PANI-MWCNT-APTES is stable in the matrix containing likely interferents.

#### 3.8.6. Real Sample Analysis

As indicated in Section 2.6, the real samples used for this study are underground water and seawater. The percentage recovery method used for this study is described in the Supporting Electronic Information (Supplementary S3). The obtained result is given in Table 1.

The percentage recovery for the spiked Cd^2+^ ranged from 103.6% to 129.9%. Thus, the obtained result is satisfactory and suitable for use in real water samples.

#### 3.8.7. Comparison with Literature

Table 2 presents previous studies on the detection of Cd^2+^ using different methods. Compared with the earlier reported method, the current study compares very well with the literature and even outperformed many. Significant progress on Cd^2+^ ion assays is the stability of PANI-MWCNT-APTES-modified GCE towards the Cd^2+^ reduction process. The typical setback in metal ion detection is electrode fouling, which makes the substrate (coated material) on the GCE have an unstable current response. However, it is suggested that crosslinking of MWCNT to the PANI-APTES matrix could have contributed to the anti-fouling nature of the PANI-MWCNT-APTES substrate.

## 4. Conclusions

The current study focused on detecting Cd^2+^ ions in water using a newly synthesized composite based on PANI-MWCNT-APTES. The composite was synthesized by the wet chemical method, and its successful synthesis, including XRD, XPS, FESEM, TGA-DTA, FTIR, and CV. The synthesized PANI-MWCNT-APTES was applied for electrochemical sensing of Cd using linear sweep voltammetry. The sensor gave a linear response to Cd^2+^ ions in the range of 0.05 µM to 50 µM with a limit of detection of 15.5 nM. It also displayed high sensitivity and stability. In addition, the developed sensor based on a PANI-MWCNT-APTES composite exhibited fast response time, and its current responses were not affected by likely interferents. It was also used satisfactorily for Cd^2+^ ion determination in real water samples. Therefore, this study provides a cheap and effective method of Cd^2+^ ion detection in the environment for human health safety and environmental pollution prevention.

## Figures and Tables

**Table 1:** Results for determining Cd (II) in real water samples.

Sample	Added (μM)	Found (μM)	Bias	Recovery (%)	RSD (%)
**Underground water**	0	0	-	-	-
	10	10.58 ± 0.14	0.58	105.8	1.3
	50	51.84 ± 0.13	1.84	103.7	0.25
**Seawater**	0	0	-	-	-
	10	10.36 ± 0.08	0.36	103.6	0.77
	50	64.49 ± 0.18	14.49	129.9	0.28

**Table 2:** Comparison of the developed method in this study with the selected existing methods.

Electrode/Substrate	Methode	Linear Range (LR) µM	Limit of Detection (LOD) µM	Ref.
Catechol-dithiol	DPV	15–35	4.5	[53]
Ag_2_S quantum dots	Fluorescence	1.0–40	0.55	[54]
CdSe quantum dots	Flourescence	1.0–22	0.32	[55]
CdTe composite	Flourescence	1.3–25	0.5	[56]
2,6-dimercaptopurine	Colorimetry	84–336	3.66	[57]
Chalcon carboxylic acid-AgNPs	Colorimetry	0.227–3.18	0.13	[58]
SnO_2_/Nafion/Au electrode	CV	44.5-400	4.4	[59]
Chitosan/carbon nanotubes modified GCE	SWASV	13.3–36	6.5	[60]
Cd-IIP-MCPE	DPASV	0.018–1.8	0.03	[61]
VMSF/ITO	DPV	1.0–20	0.23	[62]
BiFE SPE	SWASV	0.99–12.0	0.53	[63]
AuNP/Ru[(NH3)6]^3+^/modified GCE	ASV	2.66–6.12	1.77	[64]
CoPC/GCE	DPSV	0–100	0.34	[65]
Back-to-back 6B pencil electrode	ASV	0.87–3.33	2.2	[66]
PANI-MWCNT-APTES-GCE	LSV	0.05–50	0.0155	This Work

## Data Availability

Not applicable.

## Supplementary Materials

The following are available online at https://www.mdpi.com/article/10.3390/membranes11110853/s1, Figure S1:EIS spectrum for PANI-MWCNT-APTES; Figure S2: EIS spectrum for bare GCE, Table S1 The results of electrical conductivity measurement.
