# Supplementary material for: Development of Cd (II) Ion Probe Based on Novel Polyaniline-Multiwalled Carbon Nanotube-3-aminopropyltriethoxylsilane Composite
Source: Membranes (Basel). 2021 Nov 4;11(11):853. doi: 10.3390/membranes11110853 (PMC8619428; doi:10.3390/membranes11110853)
Supplement: Supplementary file 1 [file membranes-11-00853-s001.zip › membranes-1399125-supplementary.pdf]

membranes

Article

# Development of Cd (II) Ion Probe Based on Novel Polyaniline-Multiwalled Carbon Nanotube-3-Aminopropyltriethoxylsilane Composite

**Raja S. Alruwais [1,2], Waheed A. Adeosun [1,3], Amjad E. Alsafrani [1,4], Hadi M. Marwani [1,3,*], Abdullah M. Asiri [1,3], Imran Khan [5], Mohammad Jawaid [6,*] and Anish Khan [1,3,*]**

1 Chemistry Department, Faculty of Science, King Abdulaziz University, Jeddah 21589, Saudi Arabia; rajaotb@gmail.com (R.S.A.); dsnwaheed1@gmail.com (W.A.A.); Amjad.chem@hotmail.com (A.E.A.); aasiri2@kau.edu.sa (A.M.A.)
2 Department of Chemistry, Faculty of Science and Humanities, Shaqra University, Dawadmi 11911, Saudi Arabia
3 Center of Excellence for Advanced Materials Research, King Abdulaziz University, Jeddah 21589, Saudi Arabia
4 University of Jeddah, College of science, Department of Chemistry, Jeddah 21589, Saudi Arabia.
5 Applied Sciences and Humanities Section, University Polytechnic, Faculty of Engineering and Technology, Aligarh Muslim University, Aligarh – 202002, India; imrannano@gmail.com
6 Laboratory of Biocomposite Technology, Institute of Tropical Forestry and Forest Products (INTROP), University Putra Malaysia, UPM, Serdang 43400, Selangor, Malaysia
* Correspondence: hmarwani@kau.edu.sa (H.M.M.); jawiad@upm.edu.my (M.J.); akrkhan@kau.edu.sa (A.K.)

**Citation:** Alruwais, R.S.; Adeosun W.A.; Alsafrani, A.E; Marwani, H.M.; Asiri, A.M.; Khan, I.; Jawaid; M.; Khan,A. Development of Cd (II) Ion Probe Based on Novel Polyaniline-Multiwalled Carbon Nano-tube-3-Aminopropyltriethoxylsilane Composite. *Membranes* **2021**, *11*, 853. https://doi.org/10.3390/membranes11110853

Academic Editors: Natalia Pismenskaya and Hsueh-Chia Chang

Received: 13 September 2021
Accepted: 28 October 2021
Published: 4 November 2021

**Publisher's Note:** MDPI stays neutral with regard to jurisdictional claims in published maps and institutional affiliations.

**Copyright:** © 2021 by the authors. Licensee MDPI, Basel, Switzerland. This article is an open access article distributed under the terms and conditions of the Creative Commons Attribution (CC BY) license (https://creativecommons.org/licenses/by/4.0/).

**Abstract:** Cadmium belongs to the group of potentially toxic metals that have high health and environmental significance. Due to its adverse effects on the environment, this study develops an effective electrochemical sensor for detecting a polyaniline-multiwalled carbon nanotube-3-aminopropyltriethoxysilane (PANI-MWCNT-APTES) substrate cast on the GCE. The as-prepared PANI-MWCNT-APTES was prepared by a wet chemical method, and its formation was investigated using several techniques. As a result, the prepared material exhibited a limit of detection of 0.015 μM for cadmium ions ($Cd^{2+}$) in the linear dynamic range of 0.05 μM to 50 μM. Furthermore, the PANI-MWCNT-APTES-modified GCE current response was stable, repeatable, reproducible, and short. In addition, PANI-MWCNT-APTES/GCE was harnessed for the first time for cadmium detection in real water samples, and the result was satisfactory. Therefore, the recorded results suggest that the newly designed PANI-MWCNT-APTES is a promising material for detecting Cd in the near future for human health and environmental protection.

**Keywords:** linear sweep voltammetry; heavy metal detection; environmental pollution; cadmium ion detection; electrochemical sensors; potential toxic metals

## S1. Electrical resistivity.

The electrical conductivity of PANI-MWCNTs/APTES was determined using the following equations. [35]

$$\rho = \rho_{\circ}/G7 \ (W/S) \quad (1)$$

$$G_7 \ /(W/S) = (2S \ / \ W) ln2 \quad (2)$$

$$\rho_{\circ} = V/I \times 2\pi \ S \quad (3)$$

$$\sigma = \frac{1}{\rho} \quad (4)$$

where $\varrho$ is the corrected resistivity (ohm-cm); $\varrho_\circ$ is the uncorrected resistivity (ohm-cm); $G_7$ (W/S) is the correction factor used for the case with no conducting bottom surface, which is a function of W, the thickness of the sample under test (cm); S is the probe spacing (cm); *I* is the current (A); *V* is the voltage (V); and $\sigma$ is the dc electrical conductivity (S $cm^{-1}$).

**Table S1.** The results of electrical conductivity measurement.

| PANI% | Electrical resistivity (Ω Cm) | Electrical conductivity (σ) |
|---|---|---|
| **3** | 2.445 | 0.4089 S/cm |
| **5** | 2.1268 | 0.470 S/cm |
| **7** | 1.63 | 0.613 S/cm |

## Electrochemical Impedance Spectroscopy

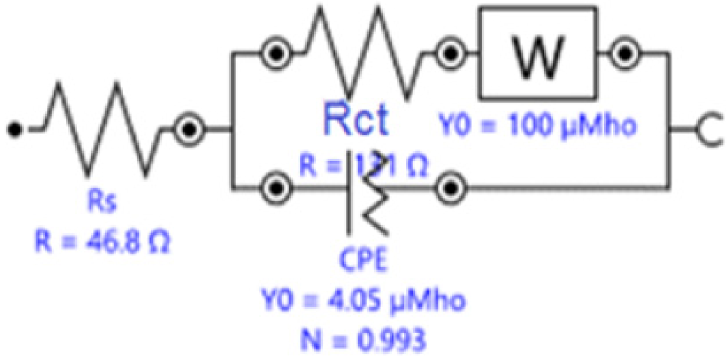


**Figure S1.** EIS spectrum for PANI-MWCNT-APTES-modified GCE.

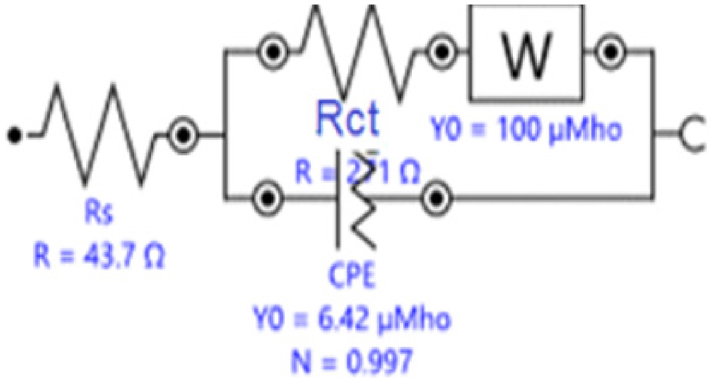


**Figure S2.** EIS spectrum for bare GCE.

## Scheme 2. Analytical performance parameters.

*Limit of Detection (LOD)*

The LOD was calculated using the following equation:

$$LOD = 3 \times Sd/b$$

where Sd is the standard deviation of the blank response and b is the calibration plot's slope.

From our results, LOD = 3 × 0.0015/ (0.29) = 0.0155 μM.

*Limit of Quantification (LOQ)*

The LOQ was determined using the following equation:

$$LOQ= 10 \times Sd/b$$

$$LOQ = 10 \times 0.0015/ (0.29) = 0.0517\ \mu M$$

*Sensitivity*

The sensitivity was calculated using the following formula:

$$Sensitivity = 0.296\ \mu A \mu M^{-1} / 0.07\ cm^{2} = 4.23\ \mu A \mu M^{-1} cm^{-2}$$
